# Fiscal decentralization in Poland: 2004-2019 municipal and city dataset

**DOI:** 10.1016/j.dib.2023.109154

**Published:** 2023-04-13

**Authors:** Rafał Trzeciakowski, Piotr Ciżkowicz, Andrzej Rzońca

**Affiliations:** Department of International Comparative Studies, Warsaw School of Economics, al. Niepodległości 162, 02-554 Warsaw, Poland

**Keywords:** Fiscal federalism, Revenue decentralization, Local public finance, Political budget cycles, Intergovernmental grants, Public investment drives

## Abstract

This dataset covers 2476–2479 Polish municipalities and cities (dependent on the year) over a period from 2004 when Poland joined the EU to the pre-COVID-19-pandemic 2019. The created 113 yearly panel variables include budgetary, electoral competitiveness, and European Union funded investment drive data. While the dataset has been created out of publicly available sources, their use requires advanced knowledge of budgetary data and their classification, as well as data gathering, merging, and clearing, which required many hours of work over a year. Fiscal variables were created out of raw data of over 25 million subcentral governments records. They were sourced from Rb27s (revenue), Rb28s (expenditure), RbNDS (balance), and RbZtd (debt) forms, which are reported quarterly by all subcentral governments to the Ministry of Finance. These data were aggregated according to the governmental budgetary classification keys into ready-to-use variables. Furthermore, these data were used to create original EU-financed local investment drives proxy variables based on large investments in general and in sports objects in particular. Moreover, subcentral electoral data from 2002, 2006, 2010, 2014, and 2018 were sourced from the National Electoral Commission, mapped, cleared, merged, and used to create original electoral competitiveness variables. This dataset can be used to model different aspects of fiscal decentralization, political budget cycles, and EU-funded investment in a large sample of local government units.

**Specifications Table**

**Table:** 

Subject:	Economics
Specific subject area:	Fiscal decentralization, local fiscal revenue, political budget cycles, public investment drives
Type of data:	Table (panel data in Excel format)
How the data were acquired:	Data were compiled from publicly available sources: Ministry of Finance (Ministerstwo Finansów), National Electoral Commission (Państwowa Komisja Wyborcza), Statistics Poland (formerly translated as the Central Statistical Office; Główny Urząd Statystyczny), Eurostat. Some additional data have been provided on request by the Ministry of Finance and National Electoral Commission.
Data format:	Analyzed and filtered secondary data. Raw data can be downloaded from the website of the Ministry of Finance (https://www.gov.pl/web/finanse/sprawozdania-budzetowe), National Electoral Commission (Elections 2002: https://wybory2002.pkw.gov.pl/wojt/index.html; Elections 2006: https://wybory2006.pkw.gov.pl/kbw/arkuszed41d.html; Elections 2010: https://wybory2010.pkw.gov.pl/Komunikaty_PKW_EN,6/Dane_W_Arkuszach_EN,8/index.html; Elections 2014: https://pkw.gov.pl/wybory-i-referenda/wybory-samorzadowe-i-referenda-lokalne/wybory-samorzadowe-w-2014ampnbspr/wyniki-wyborow/wyniki-glosowania-na-kandydatow-na-urzedy-wojta-burmistrza-prezydenta-miasta; Elections 2018: https://wybory2018.pkw.gov.pl/en/index), Statistics Poland (https://bdl.stat.gov.pl/bdl/start), Eurostat (https://ec.europa.eu/eurostat/data/database), and BeSTi@ API (https://bestia-api.mf.gov.pl/dev/index.html).
Description of data collection:	Out of raw data of over 25 million subcentral governments fiscal records from the Ministry of Finance, 80 yearly variables were created for 2476–2479 municipalities and cities (dependent on the year) for the 2004–2019 period. National Electoral Commission data from 2002, 2006, 2010, 2014 and 2018 local elections were used to create additional 6 electoral competitiveness variables. Statistics Poland data were used to convert fiscal variables into per capita terms, as well as to add additional 9 general socioeconomic variables. Eurostat data were used to convert fiscal variables into constant prices.
Data source location:	Primary data sources: • Forms Rb27s (revenue), Rb28s (expenditure), RbNDS (balance) and RbZtd (debt) which all local governments report quarterly to the Ministry of Finance. These data are published quarterly in DBF files: https://www.gov.pl/web/finanse/sprawozdania-budzetowe • Local elections candidates and results published in spreadsheets by the National Electoral Commission. ○ Elections 2002: https://wybory2002.pkw.gov.pl/wojt/index.html ○ Elections 2006: https://wybory2006.pkw.gov.pl/kbw/arkuszed41d.html? ○ Elections 2010: https://wybory2010.pkw.gov.pl/Komunikaty_PKW_EN,6/Dane_W_Arkuszach_EN,8/index.html
	○ Elections 2014: https://pkw.gov.pl/wybory-i-referenda/wybory-samorzadowe-i-referenda-lokalne/wybory-samorzadowe-w-2014ampnbspr/wyniki-wyborow/wyniki-glosowania-na-kandydatow-na-urzedy-wojta-burmistrza-prezydenta-miasta ○ Elections 2018: https://wybory2018.pkw.gov.pl/en/index • Additional 2002, 2006, 2010, and 2014 local elections candidates and results provided on request (spreadsheets and explanatory comments) by the National Electoral Commission. • Population, employment, housing etc. data from Statistics Poland BDL online database: https://bdl.stat.gov.pl/bdl/start • Price deflator from Eurostat: https://ec.europa.eu/eurostat/data/database • Constant municipal and city IDs from BeSTi@ API hosted by the Ministry of Finance: https://bestia-api.mf.gov.pl/dev/index.html • Keys for fiscal data aggregation provided on request by the Ministry of Finance.
Data accessibility:	Repository name: Mendeley Data Data identification number: 10.17632/9w2654dhhz Direct URL to data: https://data.mendeley.com/datasets/9w2654dhhz

## Value of the Data

• Revenue decentralization is the share of public revenues collected by subcentral governments. It plays a key role in the design of fiscal frameworks. It has been shown to affect public expenditure [1], fiscal balance [3], indebtedness [5], public sector productivity [4], political budget cycles [7], and GDP per capita [2]. Despite their usefulness, individual municipal and city level data are not readily available for econometric analysis. Their compilation required a complex database construction procedure, which took many workhours over a year. The resulting database consists of ready-to-use variables, e.g., fiscal data aggregated according to the Ministry of Finance aggregation keys, as well as original European-Union-financed public investment drives indicators and electoral competitiveness proxies.
• Poland is a unitary country, which makes its local governments institutionally very similar (no major differences like between American states or German Länder). In effect, Polish data makes it possible to study fiscal decentralization in a large sample of local units.
• Central European countries like Poland are unique in that they have a markedly different model of revenue decentralization than most OECD member states, relying much less on local taxes and more on taxes shared with the central government. Our data thus allows the study of a much discussed question in the literature, namely whether shared taxes should be treated as own revenue or vertical transfers [6,8].
• These data can be used by academic economists, non-governmental organizations, and governmental agencies, to inform research and policy regarding local government revenue autonomy, fiscal discipline, European-Union-financed investment drives, political budget cycles, and more. The dataset will be also of interest to international researchers who want to diversify their country-sample, but are finding it difficult to access national Polish data.
• This dataset can be used to develop and analyze revenue decentralization reforms in Poland and countries with similar fiscal frameworks, as well as draw new insights from the interplay of fiscal decentralization, fiscal sustainability, EU-financed investment, public investment drives, and political budget cycles.

## 1 Objective

While Poland is regarded as a successful case of fiscal decentralization [10,11], OECD Tax Autonomy Indicators [9] show that it is lagging behind other OECD economies in terms of subcentral government revenue autonomy. National data were collected to better understand this phenomenon, as well as draw general conclusions on the impact of local government revenue autonomy on fiscal sustainability, European-Union-financed investment drives, and political budget cycles. For this reason data collection has been limited to municipalities and cities, as these are the only local units with popularly elected executives and at least some discretion over tax rates and reliefs. The time frame has been chosen to avoid institutional breakpoints, starting in 2004 when Poland joined the European Union and ending in 2019 before the COVID-19 pandemic outbreak.

## 2 Data Description

The dataset is available in a single Excel spreadsheet (**PL_localgov_2004–2019.xlsx**). The file includes three individual Excel sheets that contain: (1) dataset that comprises 113 variables with almost 40 thousand unit-year observations, (2) descriptions for each variable in English and Polish, including MF budgetary classification codes, and sources, and (3) official Ministry of Finance budgetary data aggregation key. These sheets are summarized in Table 1.

**Table 1:** File contents of the PL_localgov_2004–2019 Dataset.

Sheet	Description
Variables	This sheet contains the dataset, which consists of 113 variables with yearly observations for 2476–2479 (dependent on the year) municipalities (urban, rural, and mixed) and cities for the 2004–2019 period. All fiscal variables are given in national currency in current prices and measured on a pure cash basis.
Descriptions	Descriptions for each variable in English and Polish, including MF budgetary classification codes, and sources
MF_aggregation_key	Aggregation key for aggregating budgetary data to official Ministry of Finance aggregates. Cleared, translated, and sourced from MF by the authors. Based on the version for Q3 2020.

The first sheet in the Excel file, **Variables**, contains 113 variables. These variables will be described below. They are clustered in Table 3 into 11 groups.

First, **IDENTIFIERS**, group contains unique unit identifiers, unit names, unit types year, and other variables that are helpful for categorizing observations.

Second, **REVENUE**, group consists of revenues aggregated according to the Ministry of Finance methodology. They disaggregate revenue into own revenue, earmarked grants, general grant – and their main categories.

Third, **EXPENDITURE**, group comprises expenditures aggregated according to the Ministry of Finance methodology. They disaggregate expenditures into capital and current expenditure, first of which includes investment expenditure, while second consists of wages and salaries, subsidies, debt servicing expenses, sureties and guarantees, social benefits, and other expenses.

Fourth, **DEBT**, group includes fiscal balance and debt variables. Debt is disaggregated into securities, credits and loans, deposits, and matured liabilities.

Fifth, **PLAN**, group encompasses planned PIT and CIT revenues for the entire year in Q1. These can be compared with actually executed PIT and CIT revenues, i.e., as a gage of uncertainty.

Sixth, **LOCAL TAXES**, group includes variables for the nine local taxes, which are (up to a maximum level set by central government) determined by municipalities and cities – namely revenues, effects of lowering the top rate, and effects of granting additional reliefs and exemptions. These can be helpful when studying revenue decentralization or tax competition.

Seventh, **FAMILY 500+**, group consists of central government grants and local government expenses for the “Family 500+” program, which is a child benefit introduced in 2016 and gradually expanded. The program was introduced in April 2016 as a monthly benefit of 500 PLN per every second, third, and subsequent child, while in families with incomes per person below 800 PLN for the first child as well. Next year its fiscal cost grew, because it was paid out for the whole year. In July 2019 the income criterion for the first child was removed. The program is so large in fiscal terms, that it may substantially affect e.g. shares of own revenue in total revenue. For this reason it may be necessary to control for it in regressions, although this data can be also used to study in which regions it is concentrated.

Eight, **NON-FISCAL**, group contains non-fiscal variables from Statistics Poland, which include population, population density, age group shares, unemployment, employment, and apartment stock. These are typically used as control variables when working with Polish local data.

Nineth, **PRICES**, group include only one variable – GDP deflator. It can be used for converting the national currency current prices fiscal variables into constant 2015 prices.

Tenth, **ELECTORAL**, group comprises variables constructed by the authors for measuring incumbency and electoral competitiveness in elections of local executives.

Eleventh, **INV_DRIVES**, group consists of variables constructed by the authors for measuring local public investment drives funded with European Union grants.

**Fig. 1:**
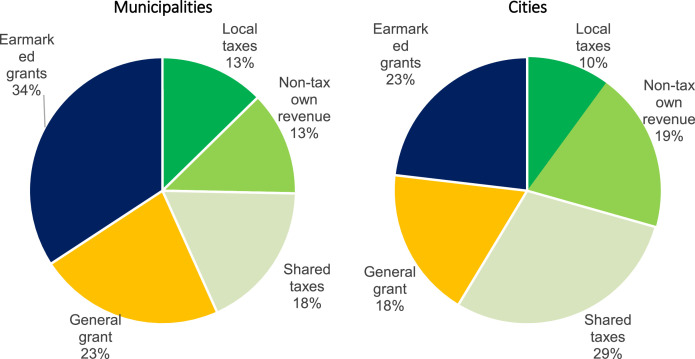
Revenue structure in 2019.

Most of the dataset consists of fiscal data sourced from Ministry of Finance databases listed in Table 2. This data is provided in national currency in current prices. It can be however easily converted into i.e. constant prices, per capita values, or percentage points of revenue. It is given on cash basis – unfortunately there is no way to convert it into accrual basis. Table 4 lists main fiscal variables and their aggregation keys. Table 5 provides basic descriptive statistics for selected fiscal variables. As an example Fig. 1 shows main municipal and city revenue category shares for 2019.

Electoral variables are constructed based on National Electoral Commission data for 2006, 2010, 2014, and 2018 local elections. They include a subcentral election year dummy, and a set of electoral competitiveness proxies. These proxies are the number of candidates running for office, whether incumbent runs for re-election, whether incumbent runs for reelection in alignment with the central government, whether incumbent is the only candidate, and whether incumbent wins in the first round of voting. Electoral competitiveness variables take the same values for entire electoral terms, i.e. if incumbent runs for re-election in 2010, dummy takes value of 1 in the years 2007–2010. Variables are described in Table 6.

Local investment drive variables are constructed based on Ministry of Finance data. Unfortunately the budgetary classification does not provide the standard statistical office division of gross fixed capital formation into buildings, vehicles, intangible assets etc. Instead, the constructed variables measure European Union funded investment drives in general and in sports objects in particular – as this is a category often invoked in popular discourse as an example of local malinvestment. Each variable is designed, so that it is triggered by particularly EU-funded large investments in general or in sports objects, accumulates with subsequent investments, and continues to drag over the current-year budget in following years. In particular:

1. The variable is triggered for a municipality or city when in year t investments from EU funds exceed X percent of revenue. This variable in year t takes the value of EU-funded investment from year t in percentage points of revenue from year t.
2. In year *t* + 1 variable takes the value of EU-funded investments from years t and *t* + 1 in percentage points of revenue from year *t* + 1.

**Fig. 2:**
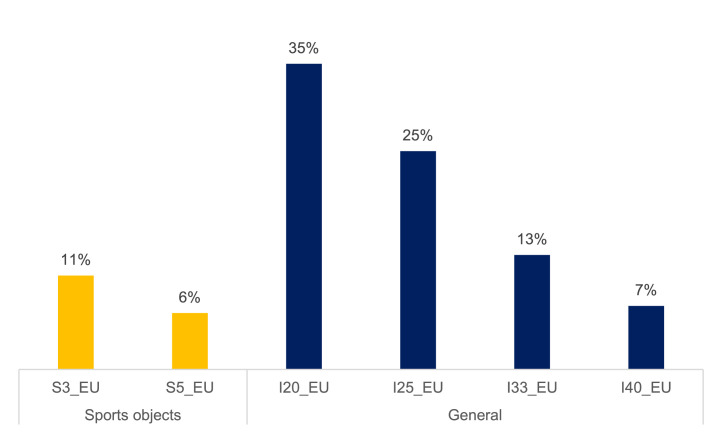
Share of year-unit observations where variable at each trigger takes values above zero.

They are provided for the following “trigger” investments: sports objects investment worth 3% and 5% of local revenue, as well as general investments worth 20%, 25%, 33%, and 40% of local revenue. Frequency of such investments in the data sample is summarized in Fig. 2.

**Table 2:** Sources of fiscal data. Ministry of Finance subcentral governments fiscal databases.

Form	Description	Description in Polish	Period (cumulative)
Rb27s	Monthly / annual report on the implementation of the subcentral governments budget revenue plan	Miesięczne / roczne sprawozdanie z wykonania planu dochodów budżetowych JST	Q1 and Q4
Rb28s	Monthly / annual report on the implementation of the subcentral governments budget expenditure plan	Miesięczne / roczne sprawozdanie z wykonania planu wydatków budżetowych JST	Q4
RbNDS	Quarterly surplus / deficit report	Kwartalne sprawozdanie o nadwyżce / deficycie	Q4
RbZtd	Quarterly Statement of Accounts Payable - Debt Titles	Kwartalne sprawozdanie o stanie zobowiązań – Tytuły dłużne	Q4

*Note:* These are selected databases, which were used to construct our dataset. The Ministry of Finance publishes them each quarter, as well as other fiscal databases at various frequencies.

**Table 3:** Groups of variables in the dataset Excel sheet.

Group	Description	No. of variables	Source
IDENTIFIERS	Unit, year, name, and type identifiers	10	Ministry of Finance, GUS Statistics Poland
REVENUE	Tax and non-tax revenue categories (official MF aggregates)	23	Ministry of Finance: Rb27s forms
EXPENDITURE	Current and capital expenditure categories (official MF aggregates)	14	Ministry of Finance: Rb28s forms
DEBT	Fiscal balance, debt categories (official MF aggregates)	7	Ministry of Finance: RbNDS, RbZtd forms
PLAN	Planned Q1 PIT and CIT revenues (official MF aggregates)	2	Ministry of Finance: Rb27s forms
LOCAL TAXES	Own revenue (official MF aggregate), local taxes (revenue, effects of lowering top rate set by the central government, effects of reliefs and exemptions granted by the unit)	32	Ministry of Finance: Rb27s forms
FAMILY 500+	Central government earmarked grants and local expenditure for the “Family 500+” program – a large child benefit introduced in 2016 and gradually expanded.	2	Ministry of Finance: Rb27s, Rb28s forms
NON-FISCAL	Population, age group shares, unemployment, employment, apartment stock	10	GUS Statistics Poland: BDL GUS
PRICES	GDP deflator (used for converting PLN current prices to PLN 2015)	1	Eurostat
ELECTORAL	Incumbency variables (constructed by the authors)	6	National Electoral Commission
INV_DRIVES	European-Union-financed local investment drives for general and sport objects investments (constructed by the authors)	6	Ministry of Finance: Rb27s, Rb28s forms

*Note:* Variables are grouped in dataset by the authors for clarity.

**Table 4:** Main fiscal variables (aggregated according to official Ministry of Finance methodology).

Variable	Description	Budgetary classification
REVENUE	Total revenue	*A* = Σ §§
A1_OWN_REV	Own revenue	A1=*A*-A16-A34
A2_CIT	CIT	Paragraph: 002
A3_PIT	PIT	Paragraph: 001
A4_AGR_TAX	Agricultural tax	Paragraph: 032
A5_R_EST_TAX	Real estate tax	Paragraph: 031
A6_FOREST_TAX	Forest tax	Paragraph: 033
A7_TRANSP_TAX	Transportation means tax	Paragraph: 034
A8_TAX_CARD	Physical entrepreneur tax paid in a tax card form	Paragraph: 035
A9_TAX_INHERITANCE	Inheritance and donation tax	Paragraph: 036
A10_TAX_CIVLEG	Tax on civil law transactions	Paragraph: 050
A11_TAX_STAMP	Stamp duty	Paragraph: 041
A12_TAX_EXPLOITATION	Minerals exploitation fee	Paragraph: 046
A13_MARKET_TAX	Market fee	Paragraph: 043
A14_PROPERTY_REV	Property revenue	Paragraph: 073,074,075,076,077,078,080,081,087
A15_OTHER_OWN_REV	Other revenue	A15=A1-A2-A3-A4-A5-A6-A7-A8-A9-A10-A11-A12-A13-A14
A16_EAR_GRANTS	Earmarked grants	A16=A17+A30+A32
A17_TARG_GRANTS	Targeted grants	A17=A18+A20+A22+A24+A26+A28
A30_200_620_GRANTS	Grants §§ 200 and 620	Paragraph: 200,620
A32_205_625_GRANTS	Grants §§ 205 and 625	Paragraph: 205,625
A34_GEN_GRANT	General grant	A34=A35+A36+A37+A38+A39+A40
A36_EDU_GRANT	Educational part of the General Grant	Paragraph: 292 and Chapter: 75,801

EXPENDITURE	EXPENDITURE	*B*=Σ§§
B1_CAP_EXP	Capital expenditure	Paragraph: 601,603,605,606,613,614,615,617,619,620,621,622,623,625,630,656,657,658,659,661,662,663,664,665,666,669,680
B2_INV_EXP	of which: investment expenditure	Paragraph: 605,606,613,614,615,617,619,620,621,622,623,625,630,656,657,658,659,661,662,663,664,665,666,669,680
B3_CUR_EXP	Current expenditure, of which:	B3=*B*-B1
B4_COMP_EXP	expenditure on wages and salaries	Paragraph: 401,402,404,405,406,407,408,409,410,411,412,417,418,478
B5_GRANT_EXP	subsidies	Paragraph: 200,205,220,226,227,231,232,233,236,241,243,248,249,250,251,252,253,254,255,256,257,258,259,262,263,264,265,266,271,272,273,278,280,281,282,283,288,290
B6_DEBT_EXP	debt servicing expenses	Paragraph: 801,804,806,807,809,811,812,813
B7_GUARA_EXP	expenditure on granting sureties and guarantees	Paragraph: 802,803
B8_BENEF_EXP	benefits for natural persons	Paragraph: 302,303,304,305,307,311,320,321,324,325,326
B9_OTHER_EXP	other expenses	B9=B3-B4-B5-B6-B7-B8

BALANCE	Surplus/deficit	C = A-B

DEBT	Debt	E

EU_EXP	Total expenditure (non-returnable foreign funds, primarily European Union budget), of which:	Financing paragraph: 1,2,5,6,7,8,9

UE1_CAP_EXP	Capital expenditure (non-returnable foreign funds, primarily European Union budget)	Paragraph: 601,603,605,606,613,614,615,617,619,620,621,622,623,625,630,656,657,658,659,661,662,663,664,665,666,669,680 and Financing paragraph: 1,2,5,6,7,8,9

EU2_CUR_EXP	Current expenditure (non-returnable foreign funds, primarily European Union budget)	UE2=UE-UE1


*Note:* The Ministry of Finance aggregation key is in Polish, English descriptions and classifications were translated by the authors. Both English and original Polish names are included in the dataset file.

**Table 5:** Main fiscal variables descriptive statistics for illustrative purposes.

Variable	Description	Obs	Mean	Std. Dev.	Min	Max
REVENUE	Total revenue	39,65	3353.7	1429.5	1350.7	53,336.7
A1_OWN_REV	Own revenue	39,65	1373.5	1228.8	231.5	49,969.0
A16_EAR_GRANTS	Earmarked grant revenue	39,65	876.8	542.8	84.5	20,194.6
A34_GEN_GRANT	General grant revenue	39,65	1103.4	345.4	207.2	3019.4
EXPENDITURE	Total expenditure	39,65	3400.7	1418.6	1375.2	68,310.2
B1_CAP_EXP	Capital expenditure	39,65	634.8	689.3	0.0	50,198.9
B3_CUR_EXP	Current expenditure	39,65	2765.9	970.1	1073.1	31,169.9
EU_EXP	European-Union-funded expenditure	39,65	241.1	392.0	0.0	17,161.5
BALANCE	Fiscal balance	39,65	−47.0	458.6	−28,089.2	32,962.4
DEBT	Public debt	39,65	808.6	765.8	0.0	37,799.2

*Note:* Table shows the number of observations, their mean, standard deviation, minimum and maximum values. The number of observations is given in thousands and is similar for all fiscal variables. Other statistics are given in national currency in current prices.

**Table 6:** Municipal and City executive main electoral incumbency variables.

	Election year			
	2006	2010	2014	2018
Municipalities				
Average number of candidates	3,2	3,1	3,2	3,2
Percentage of incumbents running for reelection	88%	90%	90%	85%
Percentage of incumbents running for reelection who are aligned with the central government	3%	24%	25%	10%
Percentage of jurisdictions where the incumbent is the only candidate	11%	12%	10%	13%
Percentage of units where incumbent won in the first-round of voting	55%	59%	52%	59%

Cities				
Average number of candidates	6,2	5,7	6,3	6,4
Percentage of incumbents running for reelection	86%	92%	88%	83%
Percentage of incumbents running for reelection who are aligned with the central government	9%	12%	20%	6%
Percentage of jurisdictions where the incumbent is the only candidate	0%	0%	0%	0%
Percentage of units where incumbent won in the first-round of voting	46%	49%	27%	48%

*Note:* This is a summary of the corresponding dummy variables in the dataset.

## 3 Experimental Design, Materials and Methods

The data were compiled following a review of local fiscal data provided by the Ministry of Finance, local election data provided by the National Electoral Commission, and general local data provided by Statistics Poland Local Data Bank (Bank Danych Lokalnych, BDL) database. Below, firstly, primary data sources characteristics are described. Secondly, dataset construction procedure from the primary data sources is outlined.

### 3.1 Primary Data Sources Characteristics

*Forms Rb27s (revenue), Rb28s (expenditure), RbNDS (balance), and RbZtd (debt) which all local governments report quarterly to the Ministry of Finance.* There are much more various data forms submitted to the Ministry of Finance by subcentral governments of various tiers with different frequencies and periods of availability, so these all had to be mapped before the four forms finally used were identified. Ministry of Finance employees were consulted for assistance, as there is little explanatory data that is publicly available. Data were sourced from the DBF databases published quarterly on the Ministry of Finance website.^1^

Databases from these forms contain very granular fiscal data with roughly 237 types of revenues (i.e. personal income tax, general grant, exchange rate gains) and 282 kinds of expenditures (i.e., various investment, compensation, and other expenditures), classified into 10 types of domestic and foreign funds sources, 34 broad sectors (i.e. tourism, public administration, family), and 878 more subsectors (i.e. foreign aid, Family 500+ childcare benefit, National Science Center). The challenge is the correct aggregation of this data, especially as their classification changes constantly, even several times a year.

There are some drawbacks to this data however. First, all data is presented on a pure cash basis, not consolidated, and not in line with ESA2010 methodology. Statistics Poland converts these to accrual basis with the algorithm $Claims_{t}-Total\;outstanding\;claims_{t-1}-Overpayments_{t-1}$ but the necessary data was not publicly available and the constructed dataset remains on a pure cash basis. Therefore the fiscal balance provided by these data is referred to as “working balance” in ESA2010 documents.

Second, these data include local government budgetary institutions only, which leaves out roughly 59,307 other units that are included in the ESA2010 local government sector in Poland. These units are^2^:

• Extra- budgetary units: budgetary establishments, own income,
• Local institutions of culture,
• Local health care institutions,
• Special purpose funds,
• Road Traffic Centres,
• Public corporations that are classified to the general government sector,
• Local hospitals with status of capital companies,
• Public schools transferred to be operated by individuals or legal persons who are not local governments,
• Public units in liquidation,
• Captive financial institutions,
• Non-profit institutions.

Third, it is not clear that databases published for the whole year by the Ministry of Finance contain final data for all units. While all subcentral units have a set date for delivering all the yearly forms, there are often many corrections to their submissions with some arriving after the deadline.

**National Electoral Commission Data.** Local elections candidates and results published (and partly provided on request) in spreadsheets by the National Electoral Commission. The challenge is connecting data from the subsequent elections, as each is presented in a different spreadsheet format. There tend to be 1–3 spreadsheets per election, with electoral rounds presented in separate files and layouts. Typos and other small errors in the results also need to be accounted for.

**Statistics Poland data.** Population, employment, housing etc. data from Statistics Poland BDL online database. This is a relatively easy to use database that can export many units and years of data into the same spreadsheet. Two major drawbacks remain however. First, Statistics Poland does not seem to account well for internal and even external migrations, which could skew the population data. Second, employment and wage data does not cover the entire labor market, i.e. employment covers 10 million people in enterprises with 10 and more workers and public sector out of roughly 16 million workers. Third, unemployment data covers only persons who register with the state as unemployed instead of the better and more commonly used Labor Forces Survey data, which is not available on the local level.

**Constant municipal and city IDs from BeSTi@ API** of the Ministry of Finance. While each quarterly set of DBF databases published on the Ministry of Finance website contains a file listing all units, these can change year-to-year with borders or reclassifications, so constant identifiers were necessary. This is a good source, as it contains all revisions of subcentral government units (“Jednostki Samorządu Terytorialnego”) with their constant ids.

**Budgetary classification codes from BeSTi@ API** of the Ministry of Finance. This is a good source, as it takes into accounts all the past methodology revisions. In particular budget classification paragraphs (“Paragrafy klasyfikacji budżetowej”, i.e. PIT revenue), financing source of budget classification paragraphs (“Finansowania Paragrafu klasyfikacji budżetowej”, i.e. non-returnable sources from European Union programs), and budget classification subsections (“Rozdziały klasyfikacji budżetowej”, i.e. “Rodzina 500+” child benefit) were downloaded.

**Keys for fiscal data aggregation provided on request by the Ministry of Finance**. These were necessary in order to follow the official classification of investment, debt servicing costs, and other aggregates.

**Eurostat data**. This is a very transparent and easy to use database, but it contains only budgetary data aggregated into general government and its subsectors in accordance with ESA2010 methodology, instead of data for each units, so only GDP deflators were used.

### 3.2 Dataset Construction Procedure

**Fig. 3:**
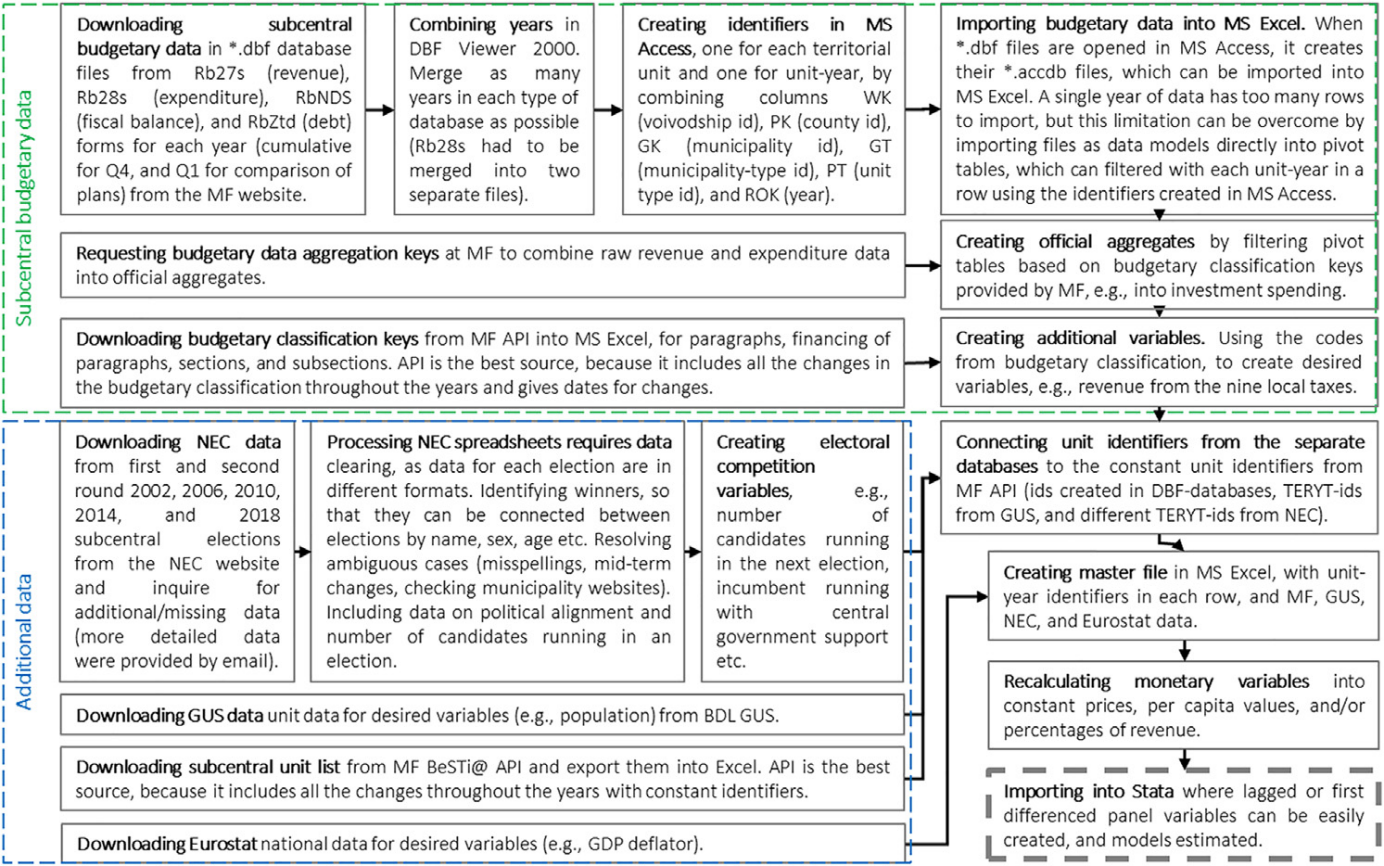
Dataset construction procedure. *MF - Ministry of Finance, NEC - National Electoral Commission, GUS - Statistics Poland. Source: Own elaboration*.

**The specific dataset construction procedure is outlined in this section and**Fig. 3**.** Yearly databases for each form category were merged using DBF Viewer 2000 (Rb28s databases were so large that they had to be merged into two separate files for 2004–2013 and 2014–2019 periods). As these observations have no unique identifiers, unit and unit-year ids were created in Microsoft Access by generating a new variable based on WK (Voivodship code), PK (County code), GK (Municipality code), GT (Municipality type), PT (County type), and ROK (year) existing variables. Subsequently the DBF files were imported into Microsoft Excel as data models (in order to avoid the number of rows limitation) for filtering and aggregation in pivot tables. Revenue and expenditure aggregates (i.e. own-source revenue, investment expenditure, debt-servicing expenditure) were constructed according to definitions used in quarterly Ministry of Finance statements on local governments budget execution, aggregation keys to which were provided on request. This aggregation was streamlined by using pivot tables, but only to a degree – often many paragraphs had to be chosen to create a single aggregate (e.g. investment expenditure). Other variables were constructed using budget classification provided through the Ministry of Finance BeSTi@ Application Programming Interface (API) for budget classification paragraphs (“Paragrafy klasyfikacji budżetowej”, i.e. PIT revenue), financing source of budget classification paragraphs (“Finansowania Paragrafu klasyfikacji budżetowej”, i.e. non-returnable sources from European Union programs), and budget classification subsections (“Rozdziały klasyfikacji budżetowej”, i.e. “Rodzina 500+” child benefit). This API was also used to download a list of local government units (“Jednostki Samorządu Terytorialnego”) with unit constant identifiers, which were used as the basis for dataset aggregating variables from all used databases.

**Electoral data has been sourced primarily from spreadsheets published on the National Electoral Commission website**. These were nevertheless supplemented by spreadsheets and explanations gathered through direct email enquiries. Microsoft Excel was used to clear, process, and join data from 2002, 2006, 2010, 2014, and 2018 municipal and city elections. Some additional web researches were necessary in order to identify incumbents when standard (name, sex, age etc.) data was unclear due to database mistakes (e.g. misspellings) or midterm changes of local executives. The NEC data made it possible to create electoral competitiveness variables based on incumbency for each municipality and city. Variables values are defined by subsequent election, e.g. the variable that shows whether incumbent is on the ballot for 2012 takes value of 1 if incumbent was on the ballot in the 2014 election and 0 otherwise. The assumption is that incumbent already expected to be on the ballot in the future and conducted policy in the whole previous term accordingly. This means that variables take identical values in 2004–2006, 2007–2010, 2011–2014, and 2015–2018 periods, while there is no data for 2019.

## Ethics statements

No ethical issues are associated with this work.

## CRediT authorship contribution statement

**Rafał Trzeciakowski:** Conceptualization, Methodology, Formal analysis, Investigation, Writing – original draft, Visualization, Funding acquisition. **Piotr Ciżkowicz:** Conceptualization, Methodology, Writing – review & editing, Supervision, Funding acquisition. **Andrzej Rzońca:** Supervision.

## Declaration of Competing Interest

The authors declare that they have no known competing financial interests or personal relationships that could have appeared to influence the work reported in this paper.

## Data Availability

Fiscal decentralization in Poland: 2004-2019 municipal and city dataset (Original data) (Mendeley Data).

## Footnotes

1 Data is also provided through an API (Application Programming Interface), which in principle makes it possible to code a program to access all MF subcentral data (from BeSTi@ database) directly and more efficiently. While it worked well for downloading small subsets of data, it was very slow (downloading expenditure data for municipalities and cities for all years took more than a week) and difficult to use for downloading whole databases. In fact, despite the help of two professional programmers and 10 months of attempts, including inquiries at the MF, the data downloaded in bulk through API contained errors and were unreliable.

2 List and number of units as presented in the document submitted by the Polish government to the European Commission, titled “Inventory of the methods, procedures and sources used for the compilation of deficit and debt data and the underlying government sector accounts according to ESA2010. Poland. May 2018 Update.”
